# Neighborhood Characteristics and Incident Stroke in US older adults: Evaluation in Two Nationwide Cohorts

**DOI:** 10.1093/geroni/igaf122.2723

**Published:** 2025-12-31

**Authors:** Kendra Sims, Torsten Neilands, Julene Johnson, Loni Tabb, Monika Safford, Suzanne Judd, Kirsten Bibbins-Domingo, M Maria Glymour

**Affiliations:** Boston University, Boston, Massachusetts, United States; University of California, San Francisco, San Francisco, California, United States; University of California, San Francisco, San Francisco, California, United States; Drexel University, Philadelphia, Pennsylvania, United States; Weill Cornell Medicine, New York, New York, United States; The University of Alabama at Birmingham, Birmingham, Alabama, United States; Journal of the American Medical Association, Chicago, Illinois, United States; Boston University, Boston, Massachusetts, United States

## Abstract

Research linking adverse neighborhood context with stroke disparities may reflect publication bias for chance associations. We compared results from the REasons for Geographic and Racial Differences in Stroke (REGARDS, n = 25,125, aged ≥ 45 years, 41% Non-Hispanic Black, 55% Stroke Belt; 2003-2020) study to the Health and Retirement Study (HRS, n = 13,197, aged > 50 years, 15% Non-Hispanic Black, 18% Stroke Belt; 2004-2020). We estimated Cox models predicting stroke for 51 American Community Survey (ACS) census tract variables, evaluating inter-cohort consistency of main, race- and region-stratified estimates. Follow-up in REGARDS (median=12.1 years; IQR: 6.5, 14.9) was similar to HRS (median=12.3; IQR: 8.0, 15.8). Cumulative stroke incidence was lower in REGARDS (5.8%) than HRS (13.9%). Twenty ACS variables had associations with incident stroke that differed by at least log(0.05) between cohorts. The percentage of vacant housing units for sale was associated with incident stroke among Stroke Belt participants in REGARDS (HR per SD: 0.92; 95% CI: 0.84, 1.02) and in HRS (HR per SD: 0.87; 95% CI: 0.73, 1.03); these associations were not observed among participants in the rest of the US in REGARDS (HR per SD: 1.02; 95% 0.96, 1.09, p for region interaction: 0.058) or HRS (HR per SD: 1.02; 95% 0.96, 1.09, p for region interaction: 0.078). None of the five other regional differences or any of the 11 Black-White differences replicated across cohorts. Inverse associations of vacancies with stroke replicated across two national studies, potentially implicating attractive housing markets as beneficial for older adult CVD health.

